# Posterior infundibular dissection: safety first in laparoscopic cholecystectomy

**DOI:** 10.1007/s00464-020-08281-1

**Published:** 2021-02-08

**Authors:** Mazen Iskandar, Abe Fingerhut, George Ferzli

**Affiliations:** 1 Department of Surgery, Baylor Scott and White Medical Center, Waxahachie, TX 75165 USA; 2 Section for Surgical Research, Department of Surgery, Medical University of Graz, Graz, Austria; 3 Department of General Surgery, Ruijin Hospital, Shanghai Minimally Invasive Surgery Center, Shanghai Jiao Tong University School of Medicine, Shanghai, 200025 P.R. China; 4 Centre Hospitalier Intercommunal of Poissy, 19 rue de la Libération Bat 2, 78300 Poissy, France; 5 Department of Surgery, NYU Langone Health, 65 Cromwell Avenue, Staten Island, NY 10304 USA

**Keywords:** Laparoscopic cholecystectomy, Bile duct injury, Surgical technique

## Abstract

**Background:**

Laparoscopic cholecystectomy is still fraught with bile duct injuries (BDI). A number of methods such as intra-operative cholangiography, use of indocyanine green (ICG) with infrared imaging, and the critical view of safety (CVS) have been suggested to ensure safer Laparoscopic cholecystectomy (LC).To these, we add posterior infundibular dissection as the initial operative maneuver during LC. Here, we report specific technical details of this approach developed over 30 years with no bile duct injuries and update our experience in 1402 LC.

**Methods:**

In this manuscript, we present a detailed and illustrated description of a posterior infundibular dissection as the initial approach to laparoscopic cholecystectomy (LC). This technique developed after thirty years of experience with LC and have used it routinely over the past ten years with no bile duct injury.

**Results:**

Between January of 2010 and December 2019, 1402 Laparoscopic cholecystectomies were performed using the posterior infundibular approach. Operations performed on elective basis constituted 80.3% (1122/1402) and 19.97% were emergent (280/1402). One intra-operative cholangiogram was performed after a posterior sectoral duct was identified. There was one conversion to open cholecystectomy due to bleeding. There were 4 bile leaks that were managed with endoscopic retrograde cholangio-pancreatography (ERCP). There were no bile duct injuries.

**Conclusion:**

Adopting an initial posterior mobilization of the gallbladder infundibulum lessens the need for medial and cephalad dissection to the node of Lund, allowing for a safer laparoscopic cholecystectomy. In fact the safety of the technique comes from the initial dissection of the lateral border of the infundibulum. The risk of BDI can be reduced to null as was our experience. This approach does not preclude the use of other intra-operative maneuvers or methods.

**Supplementary information:**

The online version of this article (doi:10.1007/s00464-020-08281-1) contains supplementary material, which is available to authorized users.

Generations of surgeons were trained to initiate the dissection in the Calot triangle when performing a cholecystectomy. This custom was carried over into the laparoscopic era without capitalizing on the entire benefits of the new exposure. Surgeons reverted to a top-down dissection whenever they faced a hostile hepatocystic triangle. Biliary tree injuries and bleeding remained major concerns.

Despite universal acceptance of LC and 30 years of technical and educational advances, the rate of BDI after LC historically ranged from 0.08 to 0.5 [1–3]. Two recent studies using large databases showed a BDI rate of 0.14% and 0.23% [2, 3]. For the patient, BDI increases morbidity and mortality, longer or repeat hospitalization, emotional and financial hardship, and a possible need for additional therapy for bile duct strictures [4–7]. For the surgeon, BDIs may create emotional and medico-legal issues [8–10]. Therefore, the emphasis in BDI should be on prevention rather than treatment. A number of methods such as intra-operative cholangiography, injection of Indocyanine green (ICG), infrared imaging, and the CVS have been suggested to ensure safer LC.

To these, we add posterior infundibular dissection as the initial operative maneuver during LC. Here, we report specific technical details of this approach developed over 30 years with no bile duct injury and update our experience in 1402 LC [11].

## Materials and methods

According to our institutional research board IRB policies, this research paper was exempt from IRB approval, and written consent was not needed. A series of 1402 LC’s performed from January 2010 to December 2019 by a single surgeon (GF) using posterior infundibular dissection was analyzed using both retrospective chart review and prospective patient follow-up. Patients with suspected bile duct stones underwent preoperative magnetic resonance cholangio-pancreatography (MRCP) routinely. The prospectively collected database included patient age, sex, elective, or emergency status, conversion to open, use of drains, use of MRCP or ERCP, use of intra-operative cholangiogram, and follow-up visits and course.

### Technique

#### Access and trocar placement

**Fig. 1:**
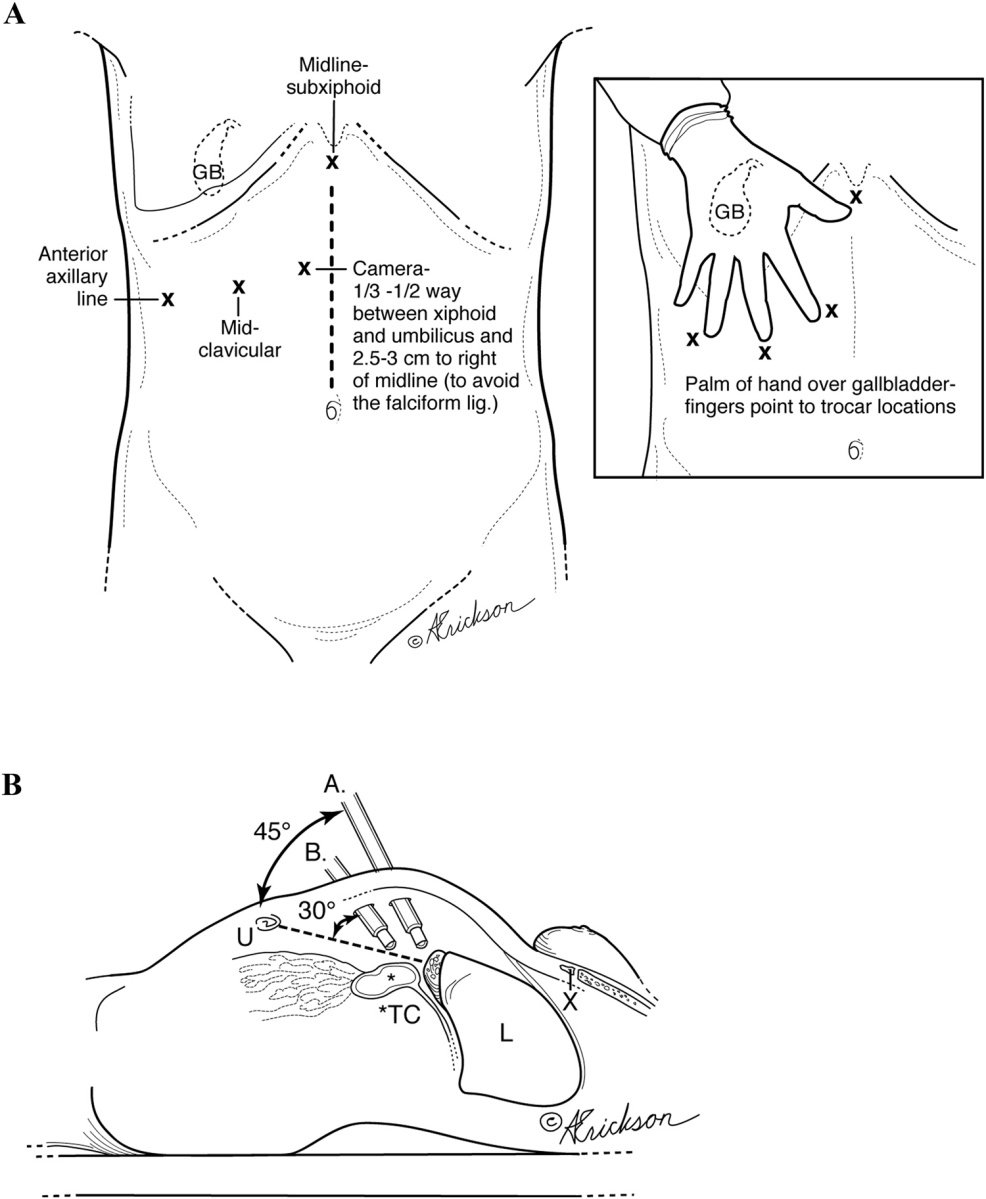
**a** Trocar placement. **b** Camera placement options for safe gallbladder dissection (3 cm to the right of midline). A. Alternate site: camera placement halfway between umbilicus and xiphoid. B. Regular camera site placement, one-third of the way between umbilicus and xiphoid. *U* umbilicus, *TC* transverse colon, *L* liver, *X* xiphoid

A Veress needle is used to create the pneumoperitoneum at the umbilicus or at Palmer’s point. Pneumoperitoneum prior to trocar insertion allows versatile trocar placement. The camera port is first placed using an optical trocar entry, at a right paramedian location 1/3–1/2, and the distance between the umbilicus and the xyphoid. A 30° scope is routinely used. Two 5-mm trocars are placed in the right anterior axillary and mid-clavicular lines, and a 10-mm trocar is placed in the subxyphoid position which will be used for gallbladder extraction [12] (Fig. 1a and b).

#### Identification of landmarks

**Fig. 2:**
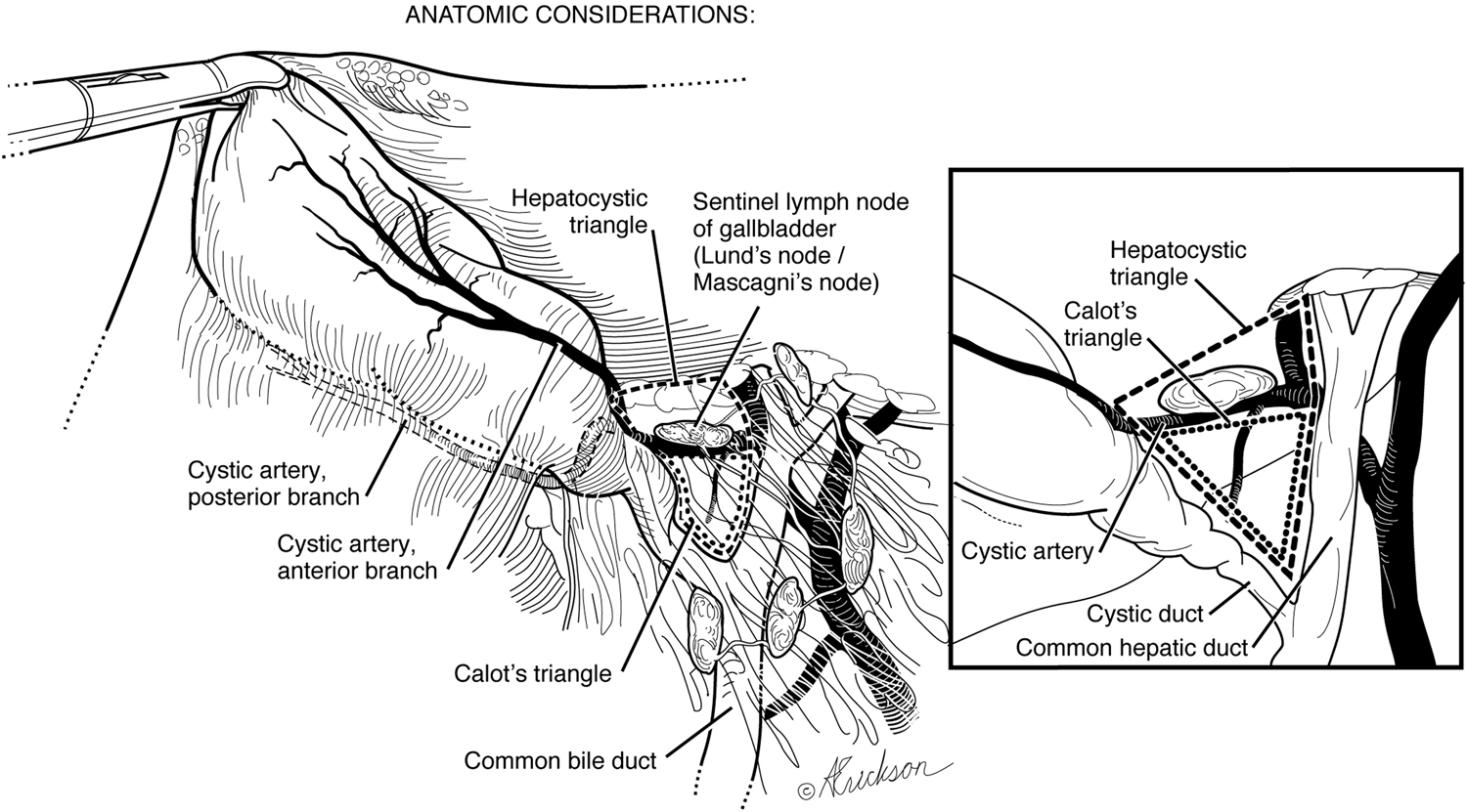
Anatomical considerations

We first visualize the area of the common bile duct, the gallbladder neck, and Lund’s or Mascagni’s node often referred to erroneously as Calot’s node [13]. This is an important landmark reliably located superior to the cystic duct, lateral to the common or right hepatic duct, and anterior to the cystic artery. Frequently, the anterior surface of the lower third of the common duct is identified as well (Fig. 2).

#### Dissection

**Fig. 3:**
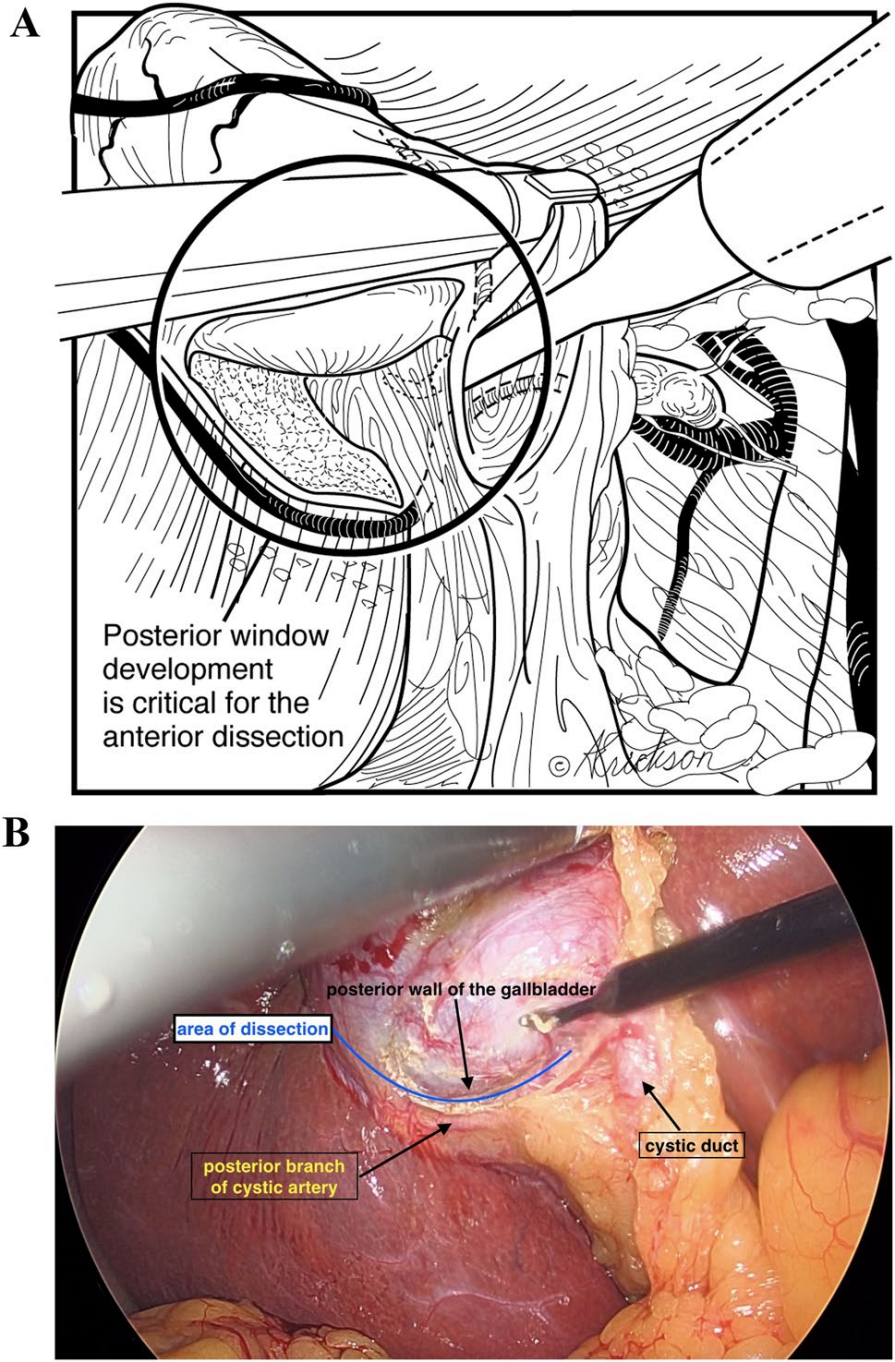
**a** Posterior dissection at the infundibulum is between the cystic artery, posterior branch, and posterior wall of the gallbladder. **b** Intra-operative view

In the case of adhesions, these are taken down in a lateral to medial fashion. Utilizing hook cautery, the peritoneum is incised anterior to Lund’s node at the infundibulum of the gallbladder. The incision is extended lateral and posterior and then superior to the dome of the gallbladder above the posterior cystic artery. With medial and cephalad retraction of the gallbladder, the avascular plane between the posterior cystic artery and the posterior wall of the gallbladder is dissected (Fig. 3a and b).

**Fig. 4:**
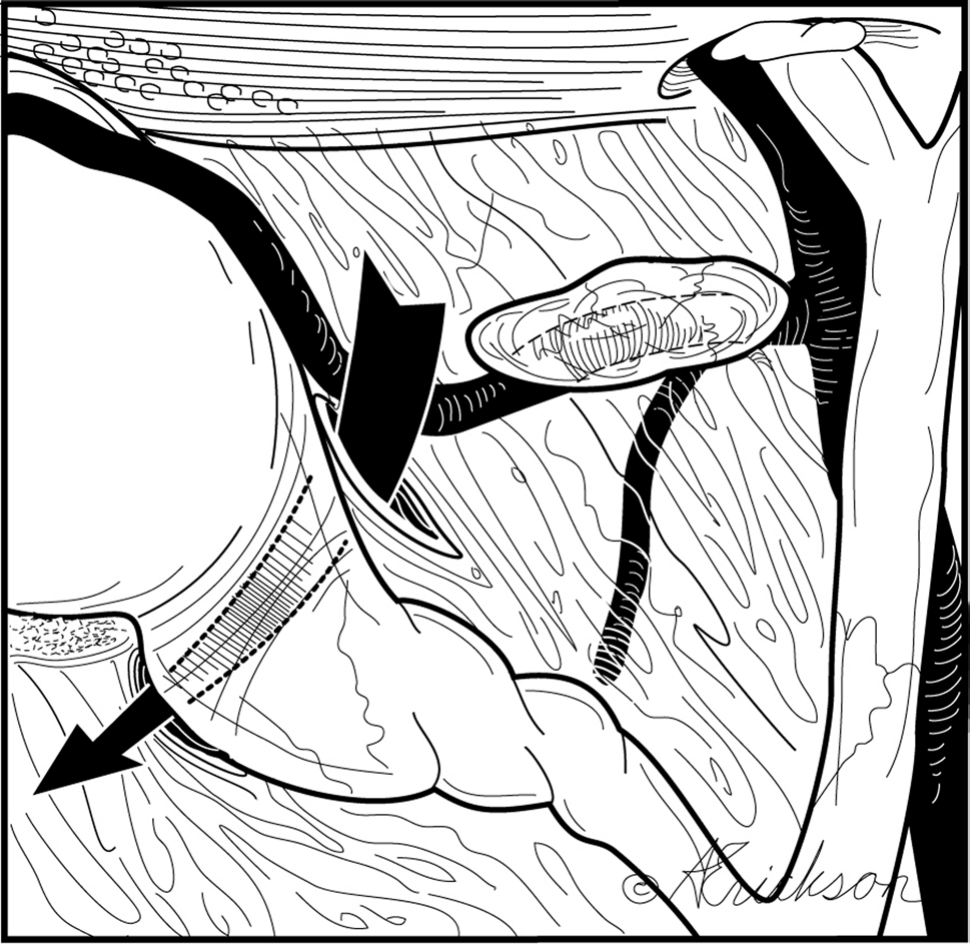
Anterior dissection is between the anterior cystic artery (after it emerges from behind the lymph node) and the gallbladder wall

Depending on the degree of inflammation and anatomy, complete or near complete circumferential dissection of the infundibulum may be accomplished. The anterior dissection is facilitated by superior and lateral traction. The peritoneal incision is extended anteriorly and laterally above the lymph node and inferior to the cystic artery. Dissection in that plane allows avoidance of the common hepatic duct, right hepatic duct, and right hepatic artery (Fig. 4).

**Fig. 5:**
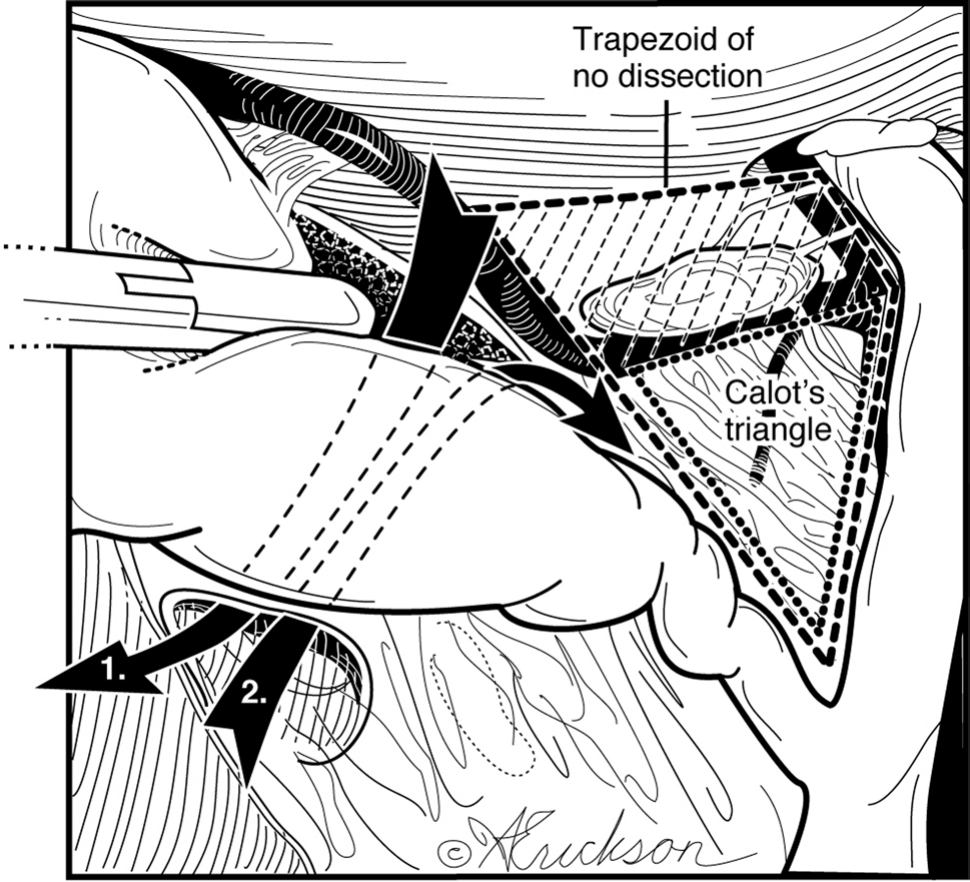
Do not operate within the trapezoidal, shaded area bound medially by the common hepatic duct, inferiorly by the sentinel lymph node of Lund, laterally by the anterior cystic artery after it emerges from behind the node of Lund and superiorly by the inferior margin of the liver. Trapezoid of no dissection = hepatocystic triangle − Calot’s triangle

The dissection between the cystic duct and cystic artery is next and allows clear visualization of the cystic duct (Fig. 5). Since most of the dissection has been done posteriorly, this is completed by a little blunt and/or sharp dissection between the posterior wall of the gallbladder and the cystic artery. The dissection proceeds in the avascular plane between the posterior wall of the gallbladder and the anterior cystic artery, dropping the artery posteriorly. No dissection is performed in the trapezoid bound medially by the common hepatic duct, inferiorly by the node of Lund, laterally by the anterior cystic artery, and superiorly by the inferior margin of the liver. This implies no skeletonization of the cystic artery. Rather, the cystic artery is clipped as it emerges from behind the node of Lund.

**Fig. 6:**
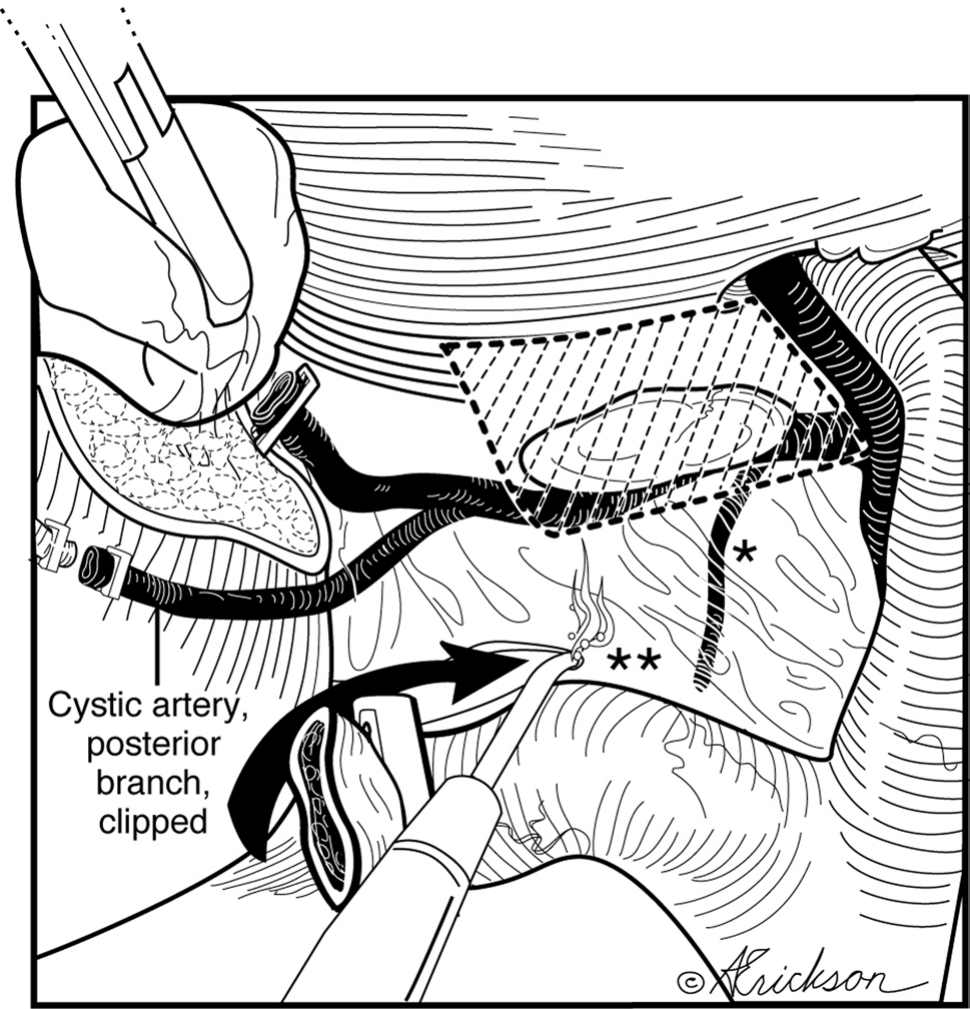
Preserved sentinel lymph node posteriorly protects the proximal aspect of the cystic artery; the medial aspect of the node points to the common bile duct. *Small arterial branch warns of the proximity of the hepatic duct. **As the dissection proceeds medially, hug the margin of the cystic duct to avoid injury due to aberrant anatomy

**Fig. 7:**
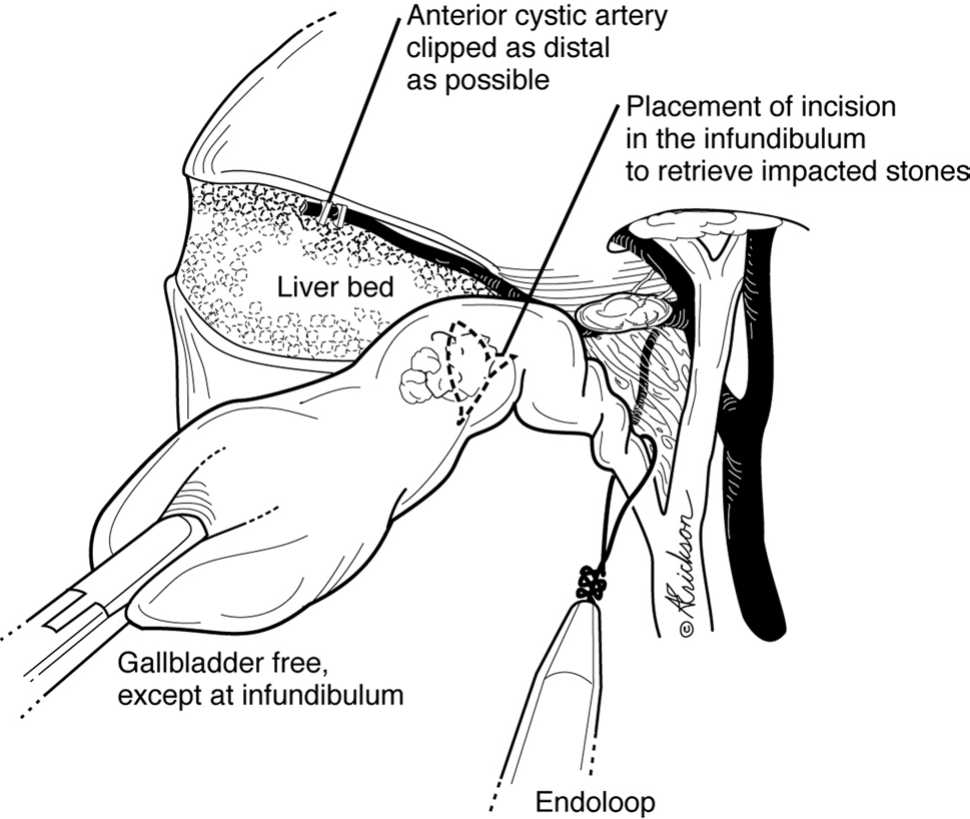
Management of impacted stone or dilated cystic duct

**Fig. 8:**
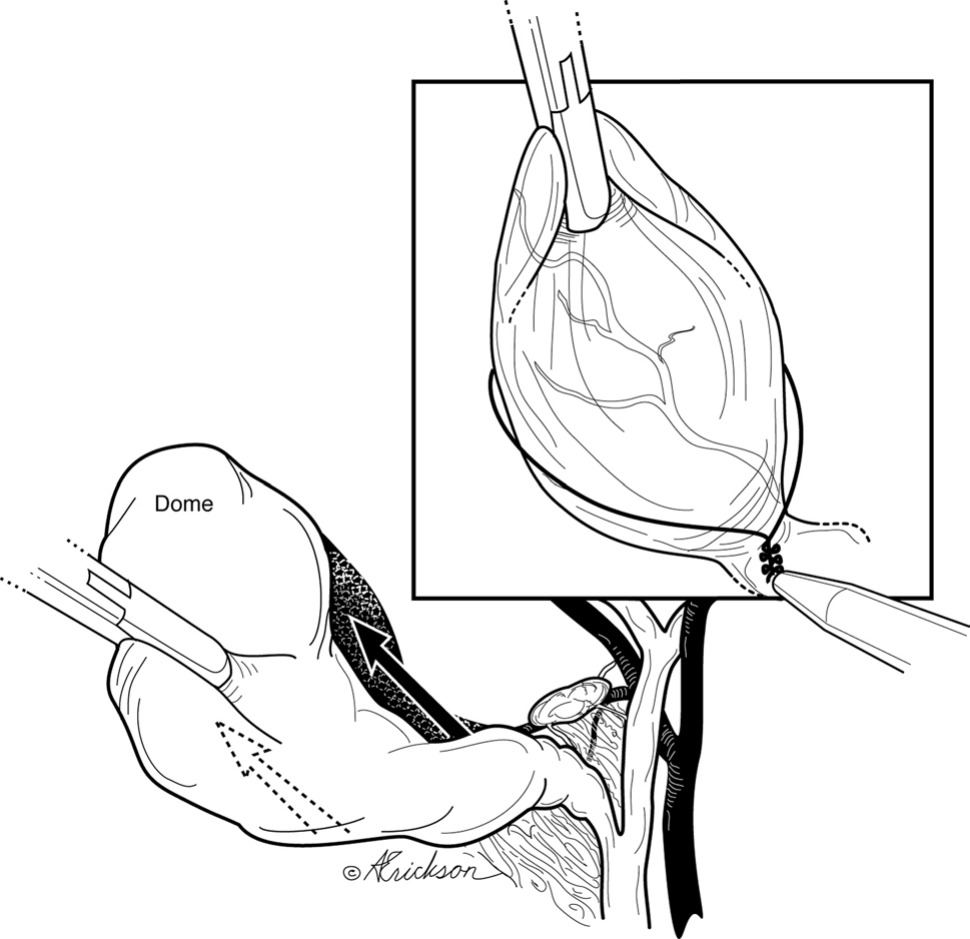
Dissection (arrows) can proceed cephalad to the dome after creation of posterior window in case of stone impaction or fibrosis at neck

The cystic duct is then clipped and divided (Fig. 6). We prefer to clip the posterior branch of the cystic artery to avoid bleeding during dissection. The remainder of the attached gallbladder is then dissected from the liver and extracted. When there is an impacted cystic duct stone that requires removal, or when the cystic duct is too dilated to accommodate clips, the gallbladder dissection is completed in the avascular plane between the posterior wall of the gallbladder and the cystic artery (Fig. 7) until the gallbladder is detached from the liver bed (Fig. 8). An incision is made in the anterior infundibulum, and the stone is extracted. The duct is then ligated with an Endoloop® (Fig. 8). After extraction of the gallbladder and lowering of the intra-abdominal pressure, hemostasis from the liver bed is verified.

## Results

The age ranged from 15 to 90 years (mean 58.1) in 1402 LC. Most patients, 77% (1080/1402), were female. Laparoscopic cholecystectomies performed on elective basis constituted 80.3% (1122/1402) and 19.97% were emergent (280/1402). Subtotal cholecystectomy was performed in 15/1402 patients (1.06%) with gangrenous cholecystitis and contracted “thumbnail” gallbladders. There were five patients with Mirizzi’s syndrome and six patients with cirrhosis. There was suspicion for bile duct stones in 18% (253/1402) of the patients where MRCP was performed. Out of those, 11% (28/253) were positive for bile duct stones and were treated with pre-operative ERCP. One intra-operative cholangiogram was performed after a posterior sectoral duct was identified. There was one conversion to open cholecystectomy due to bleeding from the liver bed. There were 4 bile leaks from the cystic duct stump that were managed with ERCP. There were no bile duct injuries.

## Discussion

Laparoscopic cholecystectomy can be very challenging depending on the severity of inflammation. Based upon the Tokyo guidelines for acute cholecystitis, there are three types of disease: mild, moderate, and severe. As the severity of the disease worsens, the surgical difficulty increases as does the risk of bile duct injury [14]. The most common cause of BDI is misidentification of the common bile duct for the cyst duct [2, 3].

Accurate anatomical identification is the most important principle during safe laparoscopic cholecystectomy. High trocar placement is one method that facilitates visualization of the triangle of Calot, common bile duct above and below the cystic duct and duodenum for proper anatomical identification. Similar to open biliary surgery, where the surgeon is positioned on the patient’s right side, a perpendicular view to the operative field is achieved. On the other hand, placement of the camera at the umbilicus leads to a more tangential view of the common bile duct especially in the obese patient where one would have to look over the transfers colon and omentum (Fig. 1b). A second method has been indirect visualization of duct anatomy by intra-operative cholangiogram or sonogram [15, 16]. In our series, 1 cholangiogram was performed to confirm the presence of a posterior sectoral duct that was identified during dissection. A third is retrograde or fundic dissection which leaves the cystic duct—common duct area intact until the gallbladder is freed from the liver bed [17]. This is often needed in the case of moderate and severe cholecystitis where dissection of the triangle of Calot can be difficult since that is the area of inflammation. In our technique, freeing the infundibulum circumferentially and creating a posterior window allow for the dissection to proceed to the dome and complete the fundic dissection (similar to the retrograde method) (Fig. 7). Bail out or alternative procedures include fenestration and reconstituting subtotal cholecystectomies or fenestrated cholecystectomy [18, 19].

**Fig. 9:**
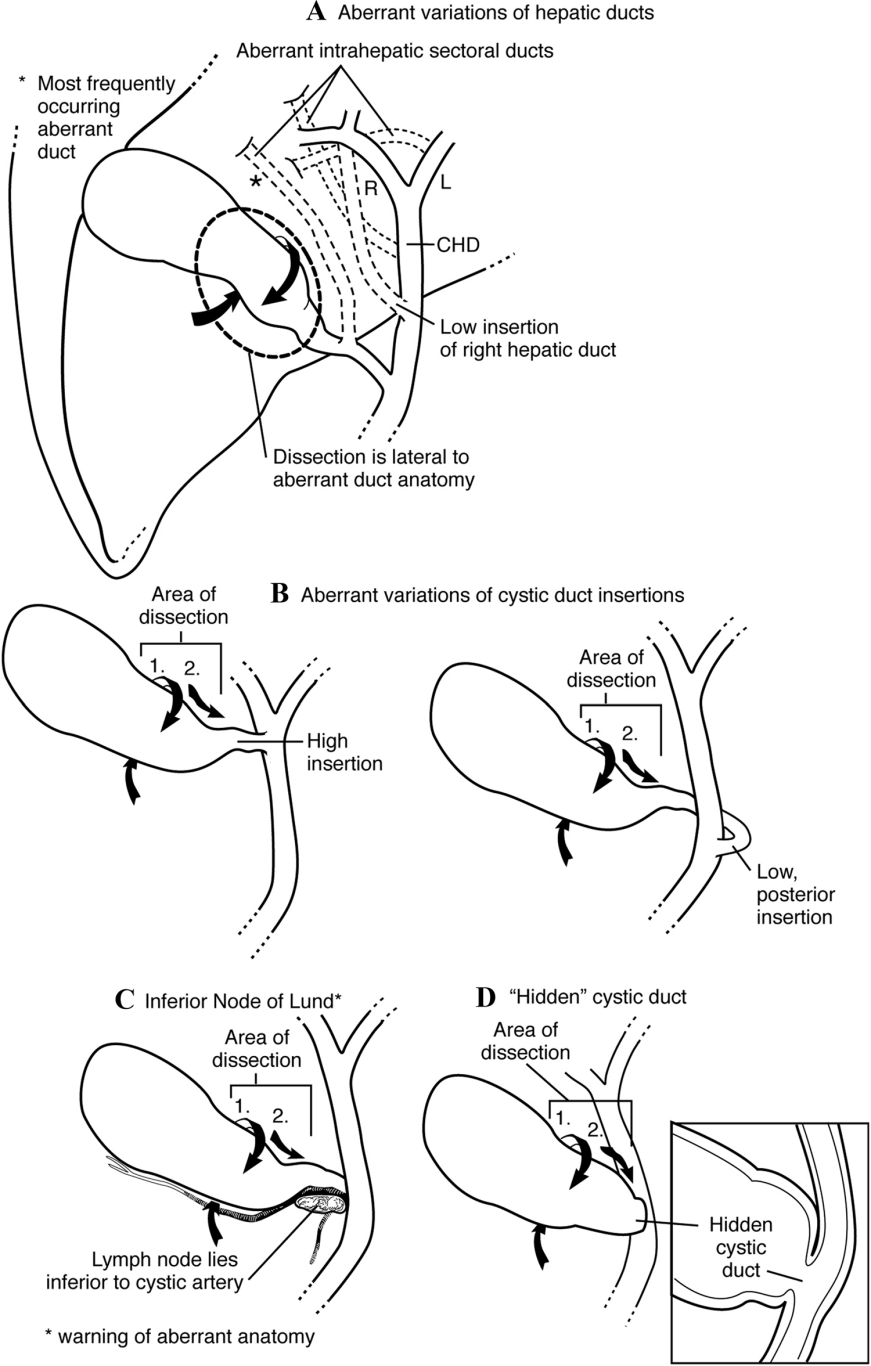
Circumferential dissection of the distal gallbladder/infundibulum must be done only after separation of the arteries (anterior and posterior) from the wall of the gallbladder. This is the key to a safe dissection from lateral to medial (towards the hepatocystic triangle). If this rule is not strictly followed, there will be potential biliary and vascular injuries in inflamed gallbladders or when aberrant anatomy is present. The same applies when the surgeon decides to take the gallbladder from top down. Separating the arterial branches from the gallbladder wall is essential for safe medial dissection

A fourth method introduced by Strasberg in 1995 for LC is “the critical view of safety.” The CVS consists of three criteria. “First, the triangle of Calot must be cleared of fat and fibrous tissue. It does not require that the common bile duct be exposed. The second requirement is that the lowest part of the gallbladder be separated from the cystic plate, the flat fibrous surface to which the non peritonealized side of the gallbladder is attached. The cystic plate, which is sometimes referred to as the liver bed of the gallbladder, is part of the plate/sheath system of the liver. The third requirement is that 2 structures, and only 2, should be seen entering the gallbladder. Once these 3 criteria have been fulfilled, CVS has been attained” [20]. The first clarification to be made is an anatomical one and relates to the definition of Calot’s triangle. Calot’s triangle as described by Calot is “an isosceles triangle with the common hepatic duct as its base, the inferior edge of the cystic duct and the superior border to the cystic artery as its sides” [21, 22]. The “modern” or current definition of the triangle of Calot differs from what Calot originally described [22]. The modern triangle Calot is better referred to as the hepatocystic triangle and is bound by the common hepatic duct medially, cystic duct caudally, and the liver edge cranially [22, 23]. CVS effectively requires skeletonization of the cystic artery and cystic duct and dissecting the cephalic aspect of the hepatocystic triangle. Our technique leaves the node of Lund intact protecting the proximal aspect of the cystic artery posterior to it. More importantly, it avoids the need to dissect the trapezoid area seen in Fig. 5a and b thereby reducing the potential injury to the common hepatic duct, right hepatic duct, and right hepatic artery. This trapezoid is actually the subtraction of the triangle of Calot from the hepatocystic triangle. To take this discussion a step further, we examined the rationale for the CVS. The CVS is meant to identify the cystic duct and artery similar to what is done in open surgery; however, it was felt that technical difficulties arise when trying to free the gallbladder completely from the cystic plate. Therefore, dissection of a third of the cystic plate provided the anatomical proof needed without the technical issues [20]. Our technique uses the same rationale and achieves the same goal again without dissection of the trapezoid and with emphasis on initial posterior dissection. Moreover, and as shown in Figs. 8 and 9, complete dissection of the gallbladder of the cystic plate replicating open surgery is achieved routinely in our technique without difficulties ligating the cystic duct and artery and again without complete dissection of the hepatocystic triangle.

An additional adjunct is systemic injection of indocyanine green and near infrared imaging [24]. None of these indirect measures substitutes for a safe technical procedure that avoids unintended division of structures during LC until the landmarks are identified [18].

Another important advantage of this technique is its applicability and success in not only cases of normal anatomy, but also in cases of aberrant anatomy. Our methodical approach consisting of initial posterior release and deliverance of the distal gallbladder/infundibulum followed by circumferential 360 degrees skeletonization of the distal gallbladder/infundibulum allows for the dissection to proceed from lateral to medial hugging circumferentially the margins of the infundibulum thereby avoiding and identifying early enough aberrant anatomic variations (Fig. 9). This approach is safe in the presence of the “hidden cystic duct” [25]. Often this stepwise approach uncoils a folded ampulla/cystic duct junction and lengthens a short appearing cystic duct.

Kirkwood al describe a middle-first approach as an option in the management of gangrenous cholecystitis [26]. In their “middle-first” approach, the dissection is started in the middle of the gallbladder to create 360° of mobilization around the gallbladder, and this is then carried towards the infundibulum to dissect the cystic ducts and artery. An extremely important point to keep in mind is that the circumferential dissection of the distal gallbladder must be done only after separation of the anterior and posterior cystic artery branches from the wall of the gallbladder. This is the key to a safe dissection from lateral to medial (towards the hepatocystic triangle). If this rule is not strictly followed, there will be potential biliary and vascular injuries in inflamed gallbladders or when aberrant anatomy is present. The same applies when the surgeon decides to rake the gallbladder from the top down. Our technique differs with the use of constant anatomical landmarks as reference points and a more systematic approach to dissection that is used routinely on all gallbladders and not just reserved to gangrenous cholecystitis.

**Fig. 10:**
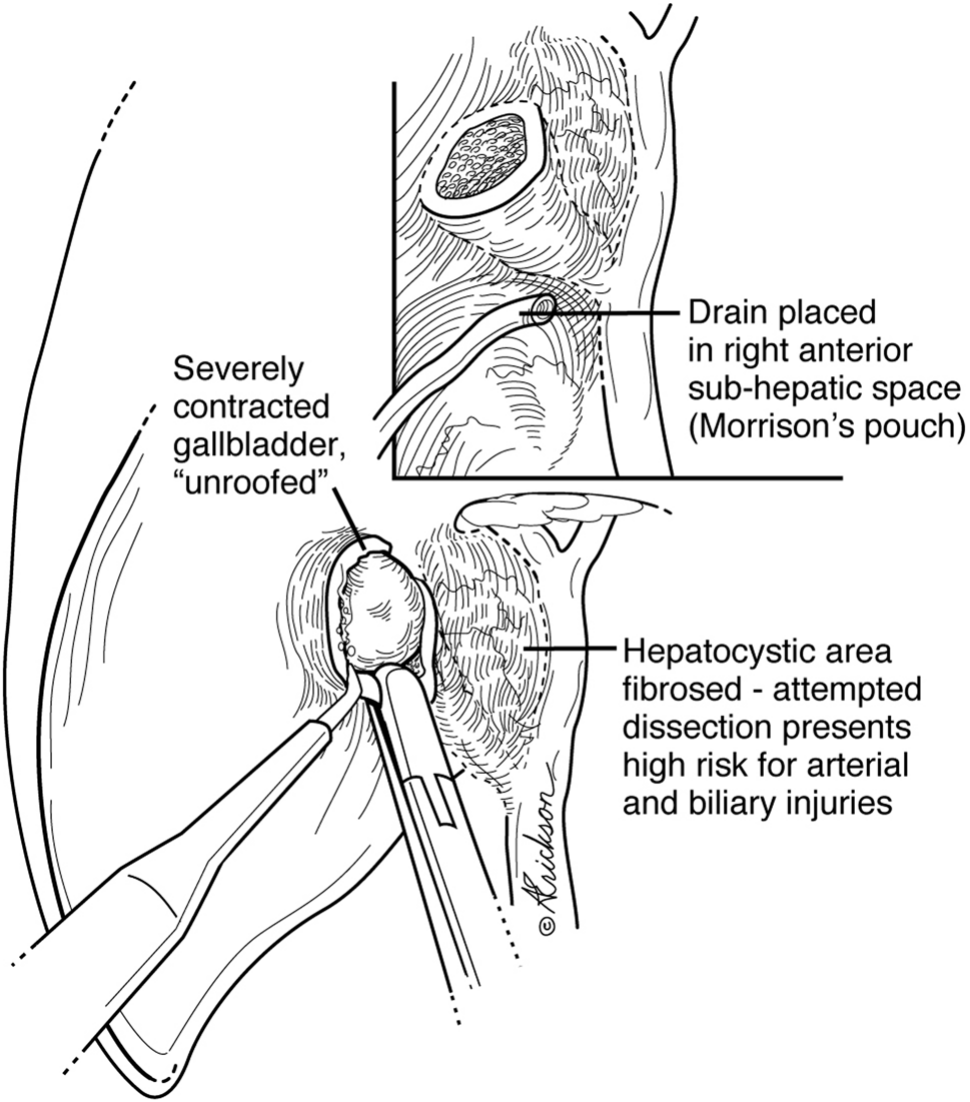
Management of contracted gallbladders

One clinical scenario where we believe that this technique is not applicable is that of the contracted or “thumbnail” gallbladder. The presence of a contracted gallbladder on preoperative ultrasound was associated with at least a higher conversion to open rate [27]. In those cases, we advocate for unroofing the gallbladder, stone extraction, and drainage (Fig. 10). Any attempts at further dissection carry a high risk for arterial and BDI’s due to fibrosis.

As shown in the results section, 18% of the patients had suspected choledocholithiasis and underwent preoperative MRCP. The authors acknowledge that preoperaritve MRCPs are less cost effective than IOCs [28]; however, most of the MRCPs were obtained prior to surgical evaluation.

By using an initial posterior approach to dissect the gallbladder and the node of Lund, there was no BDI in 1402 cases. The elements for the favorable outcomes of this approach include (a) high trocar placement to enhance visualization, (b) adopting an initial posterior approach to the dissection of the infundibulum, (c) leaving the lymph node of Lund intact, (d) avoiding the trapezoid (hepatocystic triangle minus Calot’s triangle), and (e) clipping the anterior cystic artery along its course after it has exited from behind the node of Lund.

## Conclusion

Adopting an initial posterior mobilization of the gallbladder infundibulum lessens the need for medial and cephalad dissection to the node of Lund, allowing for a safer laparoscopic cholecystectomy.

In fact the safety of the technique comes from the initial dissection of the lateral border of the infundibulum. This initial dissection avoids any aberrant ducts or vessels. The risk of BDI can be reduced to null as was our experience. This approach does not preclude the other intra-operative maneuvers or methods.

## Supplementary information

Below is the link to the electronic supplementary material.

Supplementary file1 (MP4 228925 kb)
